# The impact of spectral and temporal processing on speech recognition in children with cochlear implants

**DOI:** 10.1038/s41598-024-63932-w

**Published:** 2024-06-18

**Authors:** Andrea DeFreese, Stephen Camarata, Linsey Sunderhaus, Jourdan Holder, Katelyn Berg, Mackenzie Lighterink, René Gifford

**Affiliations:** Department of Hearing and Speech Sciences, Vanderbilt University Medical Center, 1215 21st Avenue South, Nashville, TN 37232 USA

**Keywords:** Paediatric research, Outcomes research

## Abstract

While the relationships between spectral resolution, temporal resolution, and speech recognition are well defined in adults with cochlear implants (CIs), they are not well defined for prelingually deafened children with CIs, for whom language development is ongoing. This cross-sectional study aimed to better characterize these relationships in a large cohort of prelingually deafened children with CIs (N = 47; mean age = 8.33 years) by comprehensively measuring spectral resolution thresholds (measured via spectral modulation detection), temporal resolution thresholds (measured via sinusoidal amplitude modulation detection), and speech recognition (measured via monosyllabic word recognition, vowel recognition, and sentence recognition in noise via both fixed signal-to-noise ratio (SNR) and adaptively varied SNR). Results indicated that neither spectral or temporal resolution were significantly correlated with speech recognition in quiet or noise for children with CIs. Both age and CI experience had a moderate effect on spectral resolution, with significant effects for spectral modulation detection at a modulation rate of 0.5 cyc/oct, suggesting spectral resolution may improve with maturation. Thus, it is possible we may see an emerging relationship between spectral resolution and speech perception over time for children with CIs. While further investigation into this relationship is warranted, these findings demonstrate the need for new investigations to uncover ways of improving spectral resolution for children with CIs.

## Introduction

Cochlear implant (CI) technology yields significant improvement in auditory function, speech recognition, speech production, language, reading, and overall quality of life for most recipients. Despite advances, children with CIs exhibit significant variability in speech and language development with too many recipients demonstrating suboptimal outcomes^1–6^*.* One potential source is the impoverished CI signal, which has been implicated in ongoing poorer-than-normal development across the domains of audition, speech, language, and reading^7–9^. Indeed poor spectral resolution for children with CIs, much poorer than even that exhibited by adult CI recipients, has been well documented^10–15^*.*

This age difference in spectral resolution could, in part, be that the impact of CI signal fidelity is compounded by the effects of an immature auditory system including both sensory (peripheral) and non-sensory (central and neurocognitive) factors. That is, research has shown that peripheral, within-channel spectral resolution—such as auditory filter width and critical ratio—matures in infancy^16,17^. However, behavioral measures of within-channel spectral resolution (auditory filter width^18^; on-frequency masking^19–25^) and across-channel spectral resolution (spectral ripple discrimination or spectral modulation detection^26,27^; off-frequency masking^20,22,28^) in infants and children with normal hearing (NH) are typically poorer than that observed for adult listeners, and do not reach maturation until teenage years^29–33^. This maturational trajectory, however, has not been observed for pediatric CI users^26,29,31,34,35^*.* This may be driven by fact that there is no documented evidence for changes in behavioral spectral resolution over time for children with CIs.

In adults with CIs, there is a reliable and statistically significant relationship between speech recognition and spectral resolution^10,36–40^. For children with CIs, however, the literature is mixed on whether such a relationship exists. While there exists evidence for no relationship between spectral resolution and monosyllabic word or sentence recognition^11,41^ for children with CIs, there also exists conflicting evidence for a significant relationship between spectral resolution and recognition of sentences^42^*,* vowels^26^*,* and words^43^. This conflict may be confounded by the use of abbreviated measures of spectral resolution^10,43^, assessment in the bimodal listening condition including aided acoustic hearing^43^, and relatively small sample sizes^11,26^.

Together, these differences in spectral resolution that are seen in children with CIs and the ambiguity of their relationship to speech recognition outcomes suggest that children with CIs who have prelingual onset of deafness may not depend upon spectral resolution for speech recognition in the same manner as adults. In fact, it has been demonstrated that children with hearing loss—using hearing aids and CIs—place *significantly less weight on spectral cues* (i.e. frequency rise time) than children with NH for phonemic categorization of spectrally degraded speech tokens. In contrast, children with CIs placed *greater weight on amplitude cues* (i.e. amplitude rise time)—related to temporal envelope perception—as compared to children with NH^44^. Thus, it is possible that young children with CIs are making use of different cues, such as those contained within the temporal envelope, which are well preserved with envelope-based signal coding strategies employed by current CI systems.

Interestingly, differences in cue weighting mirror differences in resolution for these cues. That is, children with CIs place less weight on spectral cues, for which they have poorer resolution than adult CI users; in contrast, they place more weight on temporal cues, for which they have been shown to exhibit better resolution than adult CI users^44,45^. Not only has there been evidence for better temporal resolution in pediatric as compared adult CI users with postlingual onset of deafness, but measures of behavioral temporal resolution have also been found to be correlated to measures of speech recognition for children with CIs^46^ as well as adults with CIs^47–51^.

Further investigation is warranted to better understand the relationships between spectral resolution, temporal resolution, and speech recognition so that we can identify the underlying mechanisms driving auditory-based speech perception in children with CIs. To better differentiate the influence of the CI signal and the immature auditory system, these relationships must be re-examined, considering both age and years of CI experience as a proxy for auditory maturation. Furthermore, to determine whether a relationship between spectral resolution and speech recognition exists for children with CIs, these constructs must be measured more systematically in a large cohort of children with CIs. Understanding these underlying mechanisms driving speech perception in children with CIs is not only necessary for theoretical purposes, but this information is critical to maximize a child’s auditory abilities in the context of both CI programming and, ultimately, speech/language/reading intervention.

The present study is the largest study to date investigating the relationships between spectral resolution, temporal resolution, and speech recognition in prelingually deafened children with CIs. Our primary hypotheses were (1) there would be no significant relationship between speech perception and either spectral or temporal resolution across all ages, (2) there would be a positive relationship between spectral resolution and age (spectral resolution improves with age), and (3) there would be no relationship between temporal resolution and age for this large group of children with CIs and congenital deafness.

## Results

### Speech recognition

**Figure 1:**
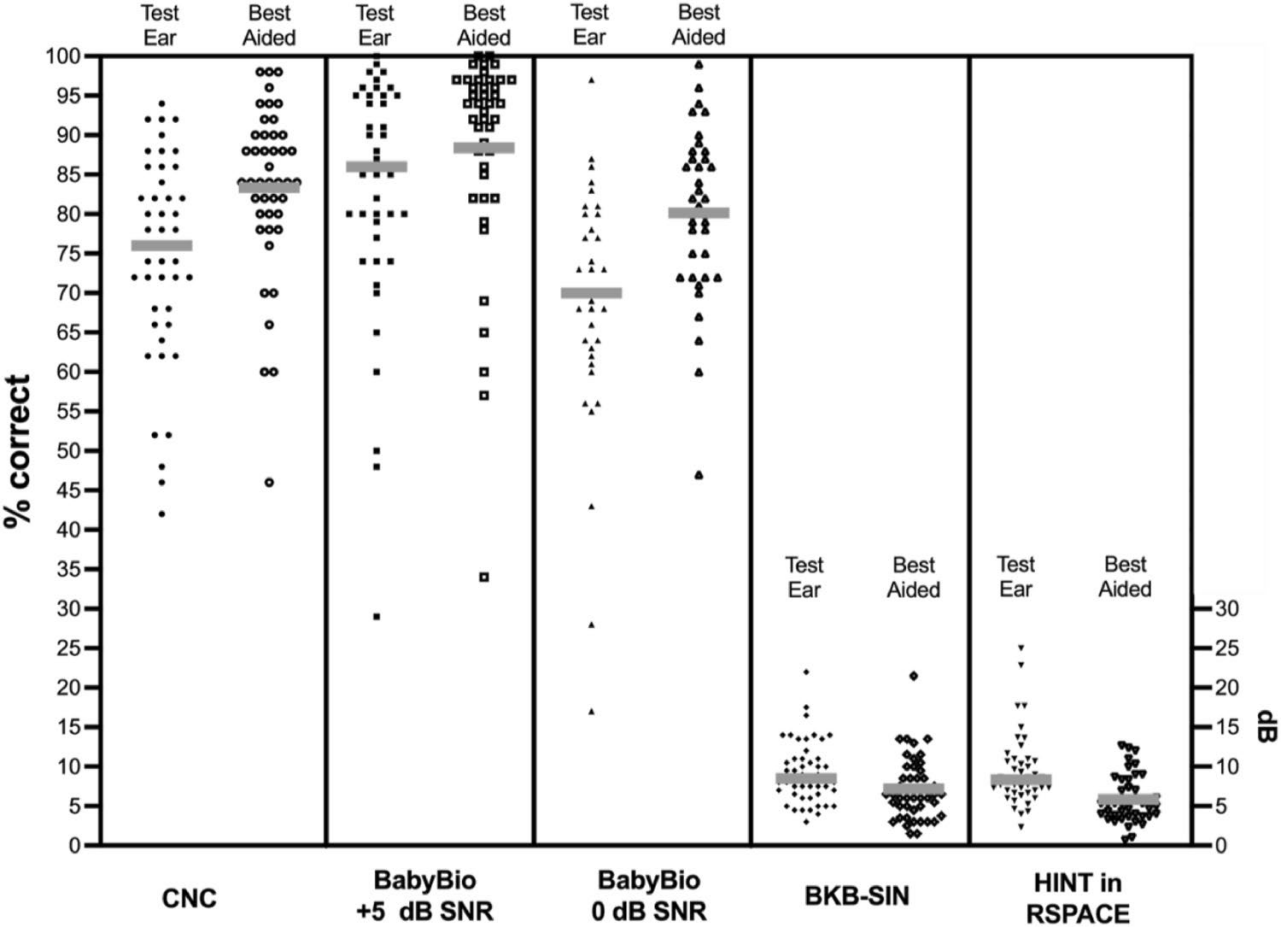
Individual (black) and mean (gray) speech recognition scores for each of the following tests: CNC word recognition, BabyBio sentence recognition at + 5 dB SNR, BabyBio sentence recognition at 0 dB SNR, BKB-SIN (SNR-50), and HINT in R-SPACE™ (SRT). For BKB-SIN and HINT, lower scores represent better performance. Scores are reported for the CI test ear alone (monaural) configuration (filled shapes) and the best aided (binaural) configuration (empty shapes).

Figure 1 displays individual and mean speech recognition scores for CNC word recognition, BabyBio sentence recognition at + 5 and/or 0 dB SNR, vowel recognition, BKB-SIN, and adaptive HINT in R-SPACE™ restaurant noise measured in the test ear (monaural) listening configuration and best aided (binaural) listening configuration. All participants completed some portion of the speech recognition testing battery; however, a few children could not complete the full battery due to time, attention, and/or compliance issues, and children whose speech recognition in quiet was sufficiently low to preclude speech in noise testing. Mean monosyllabic CNC word recognition was 75% in the test ear (*N* = 47) and 83% in the best aided configuration (*N* = 44). Mean BabyBio sentence recognition was 83% at + 5 dB SNR (*N* = 44) and 68% at 0 dB SNR (*N* = 37) in the test ear and 88% at + 5 dB SNR (*N* = 43) and 80% at 0 dB SNR (*N* = 37) in the best aided configuration. For sentence recognition in adaptive noise and fixed speech level, the mean BKB-SIN SNR-50 score was 9.26 dB in the test ear alone (*N* = 47) and 7.16 dB in the best aided configuration (*N* = 47). For sentence recognition in fixed noise with adaptive speech level, mean HINT sentence recognition with R-SPACE™ restaurant noise (S_0_N_45-315_) was 9.49 dB for the test ear alone (*N* = 43) and 5.85 dB in the best aided configuration.

### Spectral and temporal resolution

**Figure 2:**
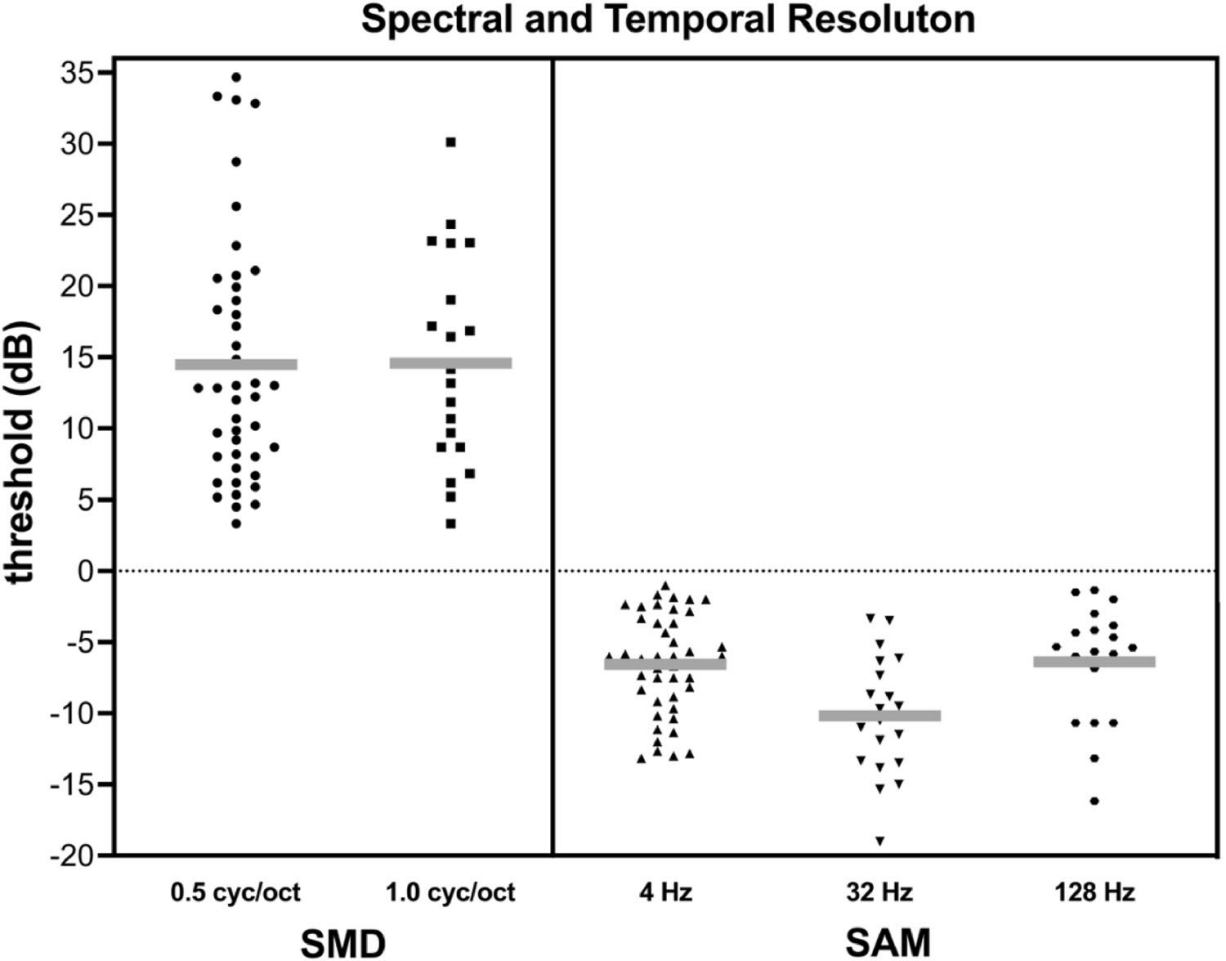
Individual (black) and mean (blue) spectral and temporal resolution thresholds. Spectral resolution was assessed using spectral modulation detection (SMD) at 0.5 cyc/oct and 1.0 cyc/oct. Temporal resolution was assessed using sinusoidal amplitude modulation (SAM) at 4 Hz, 32 Hz, and 128 Hz.

Of the 47 participants enrolled in the study, spectral resolution thresholds were successfully measured for 43 participants, as four could not complete the task which was defined as the child either not demonstrating a thorough understanding of the task and/or the child’s tracked thresholds represented spectral modulation depths ≥ 35 dB. Mean spectral modulation detection (SMD) thresholds were 14.49 dB for 0.5 cyc/oct (*N* = 43) and 14.57 dB for 1.0 cyc/oct (*N* = 20; Fig. 2). Temporal resolution thresholds via sinusoidal amplitude modulation (SAM) detection were attempted for all 47 participants, but reliable thresholds were tracked for 45 participants; that is, two participants could not complete the task even with 100% modulation corresponding to m = 1 or a logarithmically transformed threshold of 0 dB (20 log m). Mean SAM detection thresholds were − 6.56 dB for 4 Hz (*N* = 45), − 10.17 dB for 32 Hz (*N* = 20), and − 6.39 dB for 128 Hz (*N* = 20; Fig. 2).

### Relationships between speech recognition and both spectral and temporal resolution

Partial correlation analyses, controlling for duration of daily processor use (number of hours of CI use per day, i.e. datalogging, due to its known relationship with CI outcomes^52^), were used to evaluate the relationships between spectral and temporal resolution and all speech recognition measures (CNC words in quiet, BabyBio sentences at + 5 and 0 dB SNR, BKB-SIN, adaptive HINT in R-SPACE™ (S_0_N_45-315_), and vowel recognition). As mentioned above, estimates of speech understanding in noise and spectral and temporal resolution thresholds were not obtained for all participants. Similarly, we were unable to obtain reliable datalogging information, representing duration of daily processor use, for 3 of the 47 children (see “Methods” section). Therefore, the sample sizes of comparisons (seen in Table 1) are reduced. After applying Bonferroni correction for multiple comparisons, none of these relationships were found to be statistically significant. Despite this lack of significance, many did retain at least a moderate effect size^53^ (*r* > 0.3). Indeed, the highest correlation coefficients (− 0.37 to − 0.45) were observed for vowel recognition scores and both spectral and temporal resolution thresholds (see Table 1).

**Table 1:** Correlation coefficients (*R)* from partial correlations (controlling for duration of daily processor use).

	Speech recognition test					
	CNC (RAU)	BabyBio + 5 dB (RAU)	BabyBio 0 dB (RAU)	BKB-SIN (dB SNR-50)	HINT RSPACE (dB SNR)	Vowels
Spectral modulation detection						
0.5 cyc/oct	− 0.131 (*N* = 40)	− 0.348 (*N* = 40)	− 0.243 (*N* = 35)	0.174 (*N* = 40)	0.074 (*N* = 40)	− 0.427 (*N* = 24)
1.0 cyc/oct	− 0.003 (*N* = 18)	− 0.421 (*N* = 18)	− 0.333 (*N* = 18)	0.120 (*N* = 18)	0.015 (*N* = 18)	− 0.445 (*N* = 18)
Temporal modulation detection						
4 Hz	0.102 (*N* = 42)	− 0.214 (*N* = 42)	0.017 (*N* = 36)	− 0.030 (*N* = 42)	0.029 (*N* = 40)	− 0.439 (*N* = 24)
32 Hz	− 0.125 (*N* = 18)	− 0.332 (*N* = 18)	− 0.300 (*N* = 18)	0.263 (*N* = 18)	0.303 (*N* = 18)	− 0.337 (*N* = 18)
128 Hz	− 0.011 (*N* = 18)	− 0.175 (*N* = 18)	− 0.018 (*N* = 18)	0.049 (*N* = 18)	0.115 (*N* = 18)	− 0.052 (*N* = 18)

Following correction for multiple comparisons, none of the correlational analyses reached statistical significance.

### Age and CI experience

**Figure 3:**
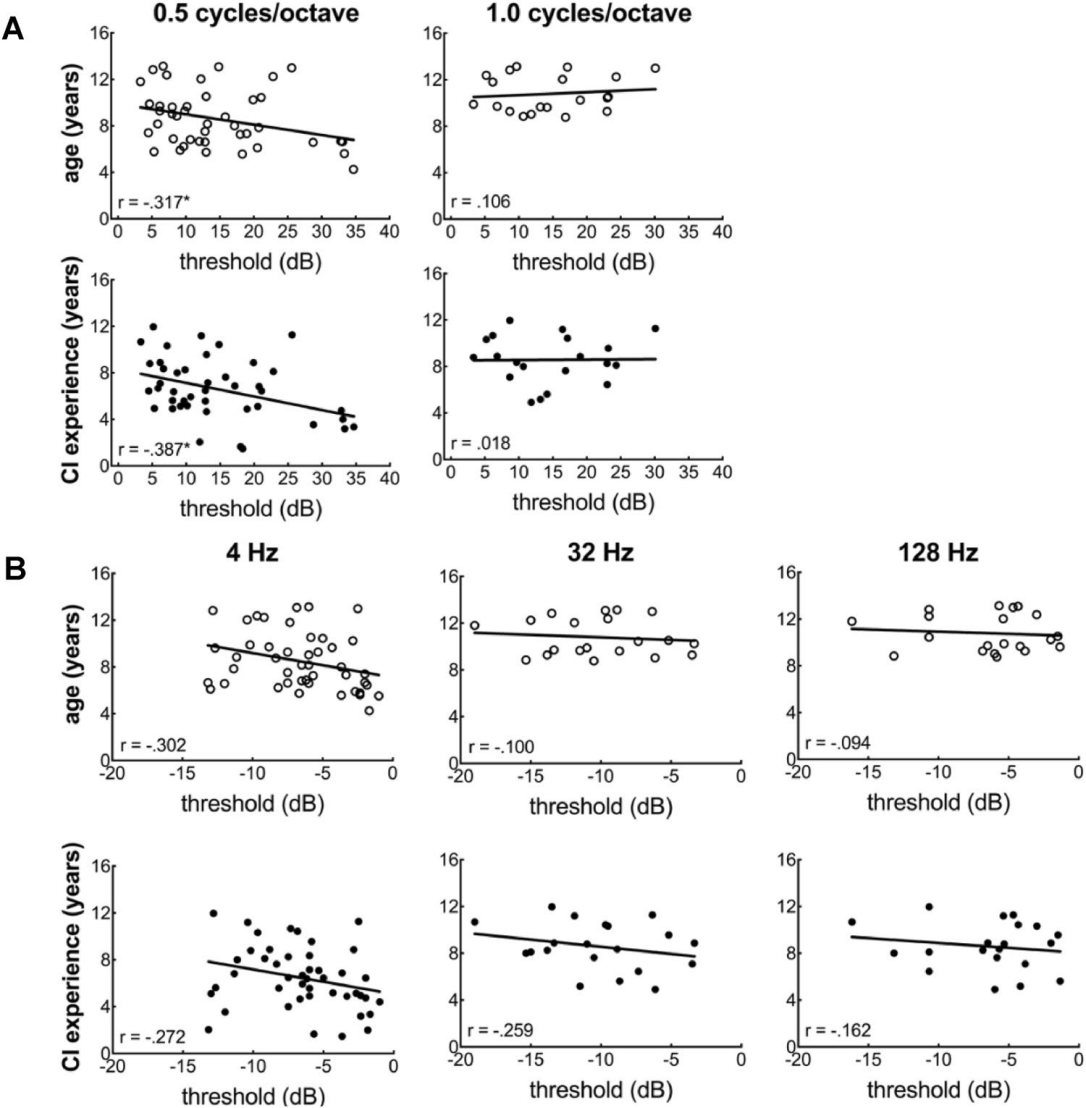
**(A**) Individual regression analyses comparing age (unfilled circles) and CI experience (filled circles) to spectral modulation detection thresholds (dB) for modulation frequencies of 0.5 cyc/oct and 1.0 cyc/oct. (**B**) Individual regression analyses comparing age (unfilled circles) and CI experience (filled circles) to temporal modulation detection thresholds (dB) for SAM rates 4, 32, and 128 Hz. *p < 0.05.

To examine the relationships between spectral and temporal resolution and both age and CI experience (years of CI use), separate partial correlation analyses were completed controlling for duration of daily processor use (mean duration of daily CI use, i.e. datalogging). After applying Bonferroni correction for multiple comparisons, none of these relationships were found to be statistically significant. Some relationships, however, were found to have a moderate effect size (r > 0.3)^53^. For spectral resolution (Fig. 3A), age and CI experience were both found to have a moderate effect on spectral modulation detection at a modulation rate of 0.5 cyc/oct (age: *r*(39) = − 0.317, *p* = 0.049; CI experience: *r*(39) = − 0.387, *p* = 0.015)), but not at 1.0 cyc/oct (age: *r*(17) = 0.106, *p* = 0.687; CI experience: *r*(17) = 0.018, *p* = 0.945)*.* For temporal resolution (Fig. 3B), the only moderate effect was observed between SAM thresholds at 4 Hz and age (*r*(41) = − 0.302, *p* = 0.055).

## Discussion

The primary aim of the present study was to investigate the relationships between speech recognition and both spectral and temporal resolution for a large group of prelingually deafened children with CIs. To account for maturation, the relationships between these measures and both chronological age and CI experience were examined.

### Speech perception in quiet and noise

In the current study, speech perception in both quiet and noise was assessed in the CI test ear alone (monaural) listening configuration and in the best aided (binaural) listening configuration. The best-aided configuration was compared to averages previously reported for children with CIs and their NH peers in effort to compare the study sample to the population at large. Overall, speech perception for the current cohort was similar to that reported in the literature for children with CIs, while remaining poorer than that of children with NH^54^. Though across-group differences were observed across all measures of speech understanding, the greatest performance differences between children with CIs (current study) and children with NH (published literature) was observed for adaptive listening tasks. For sentence recognition in adaptive noise and fixed speech levels, mean BKB-SIN SNR-50 was 7.2 dB for children with CI in the present study, compared to previous reports of 8.5 dB for children with CIs^55^ and 1.6 dB for children with NH^54^. For sentence recognition in fixed noise with an adaptive speech level (HINT in R-SPACE™), children with CIs in the current study exhibited a mean SRT of 5.9 dB SNR, which is better than the previously reported mean SRT of 10.9 dB SNR for children with CIs^56^ while also remaining significantly poorer that the mean SRT of 0.0 dB SNR observed for 25 similarly aged children with NH^56^.

Performance differences between children with CIs and NH were pervasive for word and sentence recognition with fixed presentation levels, yet smaller in magnitude as compared to adaptive speech-in-noise tasks. Mean CNC word recognition was 83% in the current study and 76% for a 620-patient sample of children with CIs^57^. Because there are no published normative data for children with NH on measures of CNC word recognition, we are unable to compare across groups on this measure. In the current study, mean BabyBio sentence recognition in noise at + 5 and 0 dB SNR was 88% and 80%, respectively. Like HINT scores, the BabyBio sentence recognition scores of the present cohort are better than the previously reported mean of 57% at + 5 dB for children with CIs^55^ while also remining significantly poorer than the means of 99% (at + 5 dB SNR) and 98% (at 0 dB SNR) reported for children with NH^54^. Consequently, it is clinically important we recognize that though children with CIs achieve high levels of speech understanding in fixed SNRs for validated measures of sentence recognition, they are still operating at a deficit as compared to their peers with NH. This is an important point guiding clinical recommendations and educational accommodations for children with CIs and is a gap our field strives to close with advancing implantable technologies and emerging hearing therapeutics.

### Spectral and temporal resolution: comparison to published data

Psychophysical estimates of spectral and temporal resolution obtained for the 47 children with CIs revealed that thresholds in both domains are generally poorer than that previously reported for children with NH. Specifically, children with CIs enrolled in the present study exhibited mean spectral modulation detection thresholds more than double the threshold reported for children with NH (7.5)^35^ and 1.5 times the threshold previously reported for adolescents with CIs (9.8)^35^ at 0.5 cyc/oct. One reason for this difference could be chronological age, given that the reference cohorts were largely adolescents with mean age of 14 years, whereas the present cohort of children with CIs ranged in age from 4.8 to 13.1 years (mean 8.3). To further investigate the effect of age, spectral resolution thresholds were compared across three age groups: 4–6, 7–9, and 10–13 years old (see Supplementary Fig. 1). When subdividing the analysis into distinct age groups, 4–6-year-olds were noted to demonstrate a poorer mean spectral resolution threshold at 0.5 cyc/oct, but with substantially more variable thresholds across the group, consistent with previous reports in this population^41,43^. No significant differences in mean temporal resolution thresholds were noted at 1.0 cyc/oct, as only 7–9- and 10–13-year-olds were tested at this frequency.

This age effect for spectral resolution has been well documented between children and adults, with or without CIs, as adults have been shown to exhibit significantly better spectral resolution than children^37,38,41^—likely the result of a combination of sensory and non-sensory factors. These across-study differences in spectral modulation detection are not, however, likely due to age at implantation, as median age at implantation for the current study (13.6 months) and the comparable literature (14.5 months)^35^ differed by less than 1 month. Therefore, while chronological age may contribute to differences in group thresholds across studies, the current results are consistent with the literature demonstrating poorer spectral resolution for school-aged children with CIs in comparison to data reported in the literature for adults with CIs as well as their peers with NH^35^. Given that this difference in spectral resolution between children with CIs and with NH persists across studies^30,35^, it likely driven by the degraded signal quality of the CI.

Temporal resolution was also found to be worse for the current sample of children with CIs when compared to published data; however, the pattern was not as clear. When compared to mean temporal resolution thresholds previously reported for 11 children with CIs (− 13.87 dB) *and* 16 adult CI users (− 10.24 dB) at a SAM rate of 100 Hz^45^, all SAM thresholds measured in the current study were poorer–even at the most comparable modulation rate (128 Hz). Though we would expect a lower (i.e. better) threshold for 4 and 32 Hz as compared to SAM rates in the 100- to 128-Hz range^58^, we observed similar thresholds across all modulation rates in the current study. The lack of a rate effect could have been driven by differences in sample sizes and ages across the three SAM rates. This relationship was further investigated by comparing temporal resolution thresholds across three age groups: 4–6, 7–9, and 10–13 years old (see Supplementary Fig. 1). When subdividing the analysis into distinct age groups, however, no significant differences in mean temporal resolution thresholds were noted at any of the modulation rates.

These across-study differences in temporal resolution could be attributed to variances in paradigm, sample sizes, and sample ages. Specifically, the sample size for the current study was more than 4 × larger than the existing literature and mean chronological ages were substantially lower (8.3 years in the present study compared to 13.7 years^45^). With this large sample size, we also observe a large degree of variability in temporal resolution thresholds, ranging from what would be predicted based on the published literature for children with CIs^59^ but also consistent with published data for similarly aged children with normal hearing^60,61^. Given this persistent variability, additional investigation is warranted including a longitudinal follow-up—which is in progress for the current project—as well as collection of temporal resolution data for a group of children and adults with NH, as well as adults with CIs, using the same experimental paradigm.

### Hypothesis 1: there would be no relationship between psychophysical estimates of spectral or temporal resolution and speech perception

After correcting for multiple comparisons and controlling for duration of daily processor use, there were no statistically significant relationships between either spectral or temporal resolution and speech recognition for all measures tested (CNC words, BabyBio sentences at + 5 and 0 dB SNR, BKB-SIN, adaptive HINT in R-SPACE™ [S_0_N_45-315_], and vowel recognition). Thus, the current results support our primary null hypothesis that there would be no relationship between speech perception in quiet or noise and either spectral or temporal resolution. These null findings are also supported by some of the published research, which indicated that speech recognition performance across a broad range of tasks for children with CIs was not related to spectral or temporal resolution as observed for adult CI users^37,38,41^. Of course, there was a limited range of ages, age at implantation, and speech recognition scores for the children in the current study; thus, additional research is needed to fully describe this relationship, ultimately allowing generalization of findings to a broader population of children with CIs.

While no relationships were statistically significant after correcting for multiple comparisons, many did retain at least a moderate effect size^53^ (*r* > 0.3). Indeed, the highest correlation coefficients (− 0.37 to − 0.45) were observed for vowel recognition scores and both spectral and temporal resolution thresholds (see Table 1). The only other speech recognition measures found to have a correlation of moderate strength with either spectral and/or temporal resolution were BabyBio and HINT sentence recognition in noise. While these relationships were not found to be statistically significant, the noteworthy effect size was further validated by support from some of the published research documenting relationships between spectral and temporal resolution and vowel recognition^26,37^ and speech in noise^42,62^.

### Hypotheses 2 & 3: there would be 2) a significant relationship between spectral resolution and age and 3) no relationship between temporal resolution and age

To further examine the effects of maturation, relationships between spectral and temporal resolution and both age at enrollment and CI experience were evaluated. For spectral resolution, while the inverse relationship between spectral modulation detection threshold and both age and CI experience was not significant after correcting for multiple comparisons, correlation coefficients (r > 0.3) were consistent with a moderate effect size^53^ for 0.5 cyc/oct (Fig. 3A). Thus, the results for 0.5 cyc/oct (Fig. 3A) were somewhat consistent with our second hypothesis that spectral resolution would be correlated with age, consistent with maturation mirroring typical development^26,29,42^. To date, however, research has not identified a direct relationship between behavioral measures of across-channel spectral resolution and chronological age for children with CIs^34,41^. For further clarification of this potentially emerging relationship, we are obtaining regular estimates of spectral resolution and speech perception over time for this group of study participants and will be able to report the trajectory of spectral resolution as a function of age for children with CIs within the context of a repeated-measures, longitudinal study.

For temporal resolution, there were no significant relationships observed with age or CI experience at any of the modulation frequencies (Fig. 3B). The only correlation coefficient consistent with a moderate effect size was observed between 4-Hz SAM and age (r = 0.302). This general lack of relationship between age and temporal resolution was consistent with our third hypothesis. However, the lack of a statistically significant relationship between temporal resolution and CI experience conflicts with previous reports. Specifically, Tuz and colleagues showed a significant correlation between psychophysical estimates of temporal resolution and duration of CI experience for 30 children with CIs; however, all children were 9–10 years of age with a broad range of ages at implantation ranging from 13 to 43 months, with a mean of 29.7 months^46^. The current sample included children over a broader range of chronological age (4.8–13.1 years) as well as age at implantation (6–67 months). Thus, it is possible that we will also observe a significant relationship between temporal resolution and duration of CI experience as we continue to follow the current sample of 47 children over time.

### Limitations

While this study included a large cohort of children with CIs, there was variability in sample sizes across all testing conditions. This is because all conditions were not completed for all participants, due to the broad range of participant ages (4.8–13.1 years), age at implantation (0.5–5.6 years), development, and cooperation. Specifically, if a participant struggled with completing the full test battery due to time restrictions, developmental ability, and/or cooperation, testing was not completed at higher modulation rates for tasks of spectral (1.0 cyc/oct) and temporal resolution (32 and 128 Hz). Different sample sizes across conditions reduces the overall power of these analyses and may therefore be impacting some of the findings. Additionally, the current sample recruited children across a broad age range but did not attempt to stratify enrollment by participant age, as this would underpower all analyses. Future studies may consider equal recruitment of children at each age to better extrapolate the specific timeline for developmental changes in spectral and temporal resolution.

## Conclusions

While there is a well-established and clear relationship between spectral resolution and speech perception for adult CI users, the findings are mixed for children with CIs. In an effort to determine the mechanisms driving speech perception in children with CIs, the relationships between spectral resolution, temporal resolution, speech recognition, age, and CI experience were examined in the present study. The current findings demonstrated the following:

1. Spectral and temporal resolution was poorer for children with CIs (measured in the current study), as compared to that reported for children with NH in extant literature^35,45^.
2. While neither spectral or temporal resolution were significantly correlated with speech recognition, both had moderate effects on vowel recognition and speech recognition in noise, suggesting these features may help aid speech understanding in difficult listening conditions.
3. Both age and CI experience had a moderate significant effect on spectral resolution (0.5 cyc/oct), but not temporal resolution, suggesting spectral resolution may improve with maturation.
4. Thus, it is possible we may see an emerging relationship between spectral resolution and speech perception over time in this group. Longitudinal experimentation and data analysis is in progress.

As a result of these findings, further investigation into ways to improve spectral resolution for children—such as via signal processing, electrical stimulus delivery, or audiologic CI programming—may in turn help improve speech recognition outcomes for children with CIs, particularly during a period of immature spectral processing.

## Methods

### Participants

Data from 47 prelingually deafened children with CIs between the age of four and a half and 13 years old (*M* = 8.33) were collected. Demographic data, including age at enrollment, age at implantation, and duration of daily processor use (i.e. datalogging) for the test ear at baseline for these participants can be found in Table 2. CI experience (in years) was calculated from the difference between age at study enrollment and age at implantation. Age and CI experience were used as variables in future analyses, whereas duration of daily processor use (average number of hours of CI use per day, i.e. datalogging) was used as a covariate in all analyses.

**Table 2:** Participant demographics including mean age at implantation, age at enrollment, and mean daily processor use; Parenthetical values represent the range for each measure.

	*N* = 47
Age at implantation (years)	*M* = 1.75 (0.50–5.58)
Age at enrollment (mean years)	*M* = 8.3 (4.75–13.1)
Duration of daily processor use (mean hours/day)	*M* = 11.8 (4.0–16.7)

All participants had bilateral sensorineural hearing loss for which they received a CI prior to the age of six (mean: 1.75 years; range: 0.50–5.58 years). Forty-three of these participants (91.4% of the sample) were bilateral CI users whereas four used a bimodal hearing configuration (CI plus contralateral hearing aid). All participants relied on auditory/oral methods as the primary mode of communication in an English-speaking household (note: one participant comes from a multilingual household, but the primary language used in the home is English). Additionally, all participants had nonverbal IQ within the age-normative range—with Leiter-3^63^ standard scores ranging from 87 to 126 (mean: 120.2)—and none had any additional disabilities that would have impacted their ability to complete the behavioral tasks including attention-deficit/hyperactivity disorder, learning disability, or any additional diagnosis impacting cognition.

Testing was completed using the child’s standard clinical CI maps, programmed using electrically evoked stapedial reflex thresholds (ESRTs) to shape the upper stimulation level profile. Lower stimulation level settings were verified to be providing CI-aided detection thresholds in the range of 15–30 dB HL to frequency-modulated pure tones (250–6000 Hz) presented in the sound field.

### Stimuli and presentation

Stimuli for spectral and temporal resolution tasks were generated using MATLAB and presented via loudspeaker at 0 degrees azimuth, 1 m from the participant seated in a sound treated booth. Prior to data collection, stimuli were calibrated at 65 dB SPL (A weighted) using a Larson Davis SoundTrack LxT sound level meter. All spectral and temporal resolution testing was completed in a unilateral, CI alone listening configuration. For the four bimodal listeners, the contralateral HA was turned off with the fully occluding earmold left in place. To maintain the monaural listening condition for the bilateral CI users, one ear was selected as the test ear. This ear was selected as the poorer performing ear for cases in which there was interaural asymmetry on clinical measures of speech perception. For children with symmetric speech perception across ears, the choice of experimental ear was made on the basis of child and parent/guardian preference. All testing was completed for this test ear alone and the sound processor for the non-test ear was removed for testing. For the speech recognition testing, however, testing was completed with this test ear alone, as a monaural listening configuration, and in a best aided (CI and contralateral ear [hearing aid or second CI]), as a binaural listening configuration. Only the monaural, CI-alone configuration was analyzed in the present study to isolate the influence of electric-only hearing. Each participant was given training with correct answer feedback prior to each of the following tasks to ensure they were able to provide reliable results. The methods and procedures outlined below were all approved by Vanderbilt University’s Institutional Review Board prior to testing (IRB #190095). All methods were performed in accordance with these guidelines and regulations. Prior to participation, informed assent was obtained from all participants (minors) and informed consent was obtained from a parent and/or legal guardian.

### Procedures

#### Spectral resolution: spectral modulation detection

Spectral resolution was assessed using a three interval, two alternative forced choice task with a 500 ms broadband carrier (125–5600 Hz). The first interval was a cue, containing a flat spectrum noise. Either the second or the third interval was a duplication of this cue, whereas the remaining interval contained the spectrally modulated stimulus. Participants were tasked with selecting which interval (two or three) contained the modulated stimulus (i.e. which was different). Responses were recorded after a participant selected a box via graphical user interface (GUI) labeled 2 or 3 for the spectrally modulated stimulus using is a touchscreen monitor. Four runs with 80 trials each were completed at each visit, two trials with a modulation frequency at 0.5 cycles/octave (cyc/oct) and two trials at 1.0 cyc/oct. While modulation frequency was fixed, modulation depth (dB) varied adaptively using a 2-down, 1-up procedure to track the 70.7% threshold^64^. Initial step size was 4 dB and then decreased to 2 dB after two reversals. Spectral modulation depth threshold (dB) was determined for each run by averaging the last six reversal points of that run. The two runs for each modulation frequency were averaged to determine reported threshold. For cases in which the thresholds for the two individual tracks were separated by more than 5 dB, we completed a third run and averaged across the three measures for a given modulation rate. For the sake of time as well as participant attention and cooperation with a large experimental protocol, participants eight years old or younger only completed this task at a modulation frequency of 0.5 cyc/oct.

#### Temporal resolution: temporal modulation detection

Temporal resolution was assessed using a three-interval forced choice task with a 500 ms broadband carrier stimulus with a bandwidth from 125 to 5600 Hz. Two of the intervals contained a flat temporal envelope and one interval (either one, two, or three) contained a sinusoidal amplitude modulation (SAM). Participants were tasked with selecting which interval contained the stimulus with SAM (i.e. which sound was different). Responses were recorded using a similar GUI on a touchscreen monitor as described above. Six runs of 80 trials each were completed at each visit, two trials with for each of the following SAM rates: 4, 32, and 128 Hz. For each trial, a temporal modulation threshold was defined as 20 log m (dB), with m as the modulation index (0–1). Similar to the spectral resolution task, temporal modulation rate was fixed, and modulation depth (dB) was varied adaptively using a 2-down, 1-up procedure to track the 70.7% threshold^64^. Initial step size was 4 dB and then decreased to 2 dB after two reversals. The two runs for each rate were averaged to determine reported threshold, with a third run included in cases for which the threshold estimates from each run were separated by 5 dB. These first two rates (4 and 32 Hz) were assessed to define the plateau of the temporal modulation transfer function, whereas the third rate (128 Hz) was assessed to define the sloping portion of the function. For the sake of time and participant attention and cooperation, children eight years old or younger only completed this task at 4 Hz.

#### Speech recognition

Speech recognition was assessed in both quiet and noise for the CI test ear alone (monaural) listening configuration and in the best aided (binaural) listening configuration. In the binaural listening condition, the children wore both the CI processor for their test ear and their hearing device (hearing aid or cochlear implant) on the contralateral ear. Monaural testing was completed for comparison to the spectral and temporal resolution thresholds measured in the present study while binaural testing was completed to compare study sample to population at large. For quiet conditions, monosyllabic word recognition was evaluated using the Consonant-Nucleus-Consonant lists^65^ and synthetic vowel recognition was evaluated using a /bVt/ format with 3 repetitions presented for each of the 13 vowels. All stimuli in quiet were presented at 60 dB SPL from a single loudspeaker at 0 degrees azimuth, 1 m from the participant seated in a sound treated booth, as prescribed by the Pediatric Minimum Speech Testing Battery for children with hearing loss^66^.

For assessments of speech in noise, testing was completed with both collocated speech and noise (S_0_N_0_) and spatially separated speech and noise using the Revitronix R-SPACE™ sound simulation system consisting of an eight-loudspeaker array placed in a circular pattern around the participant. For the collocated condition, speech stimuli were presented at 65 dB SPL from a single loudspeaker at 0 degrees azimuth, 1 m from the participant seated in a sound treated booth, as prescribed by the Pediatric Minimum Speech Testing Battery for children with hearing loss^66^. In this configuration, sentence recognition was evaluated using the pediatric AzBio sentences (BabyBio^67^) in multi-talker babble at + 5 dB SNR and 0 dB SNR for participants who scored greater than 80% at + 5 dB SNR. In this collocated listening condition, an SNR-50 was also measured using the Bamford-Kowal-Bench speech in noise (BKB-SIN)^68^ task.

For the spatially separated testing, an adaptative speech reception threshold (SRT) was obtained using Hearing in Noise Test (HINT)^69^. The R-SPACE™ restaurant noise was presented from seven of the eight loudspeakers, ranging from 45 to 315 degrees (S_0_N_45-315_). The noise had an overall level of 72 dB SPL which was the physical level measured in the original restaurant recording^70^ as described in previous CI studies with adults^71,72^ and children^56^. HINT target speech was presented at the speaker positioned at 0 degrees at a level adapted to determine the SNR for 50% correct, measured using a one-down, one-up stepping rule. Initial step size was 4 dB which decreased to 2 dB after the first two reversals. Each participant completed two trials, each with two 10-sentence lists presented in a 20-sentence sequence. For each trial, the last six presentation levels were averaged to provide a SRT with the reported SRT representing the mean of the two trials.

Since CNC words and BabyBio sentences are not adaptive measures, the scores could be impacted by floor and ceiling effects. In order to account for this these effects, the scores were converted from percent correct to rationalized arcsine units or RAU^73^ prior to all analyses.

### Statistical approach

To investigate the relationships between spectral resolution, temporal resolution, and speech recognition, partial correlation analyses were completed, controlling for duration of daily processor use (i.e. average daily CI use; datalogging). To examine the relationships between spectral resolution, temporal resolution, and both age and CI experience (total years of CI use), separate partial correlation analyses were completed, also controlling for duration of daily processor use. Given the multiple comparisons completed in these analyses, separate Bonferroni corrections were completed. Prior to all analyses, the distributions for all variables were found to be normally distributed (skewness < 2, kurtosis < 7^74^). All statistical analyses were completed in IBM SPSS Statistics Version 27.

### Institutional review board approval

Vanderbilt University Institutional Review Board; IRB# 190095; PI: Gifford.

### Supplementary Information

Supplementary Information.

## Data Availability

The datasets generated during the current study are not publicly available as they are a part of an ongoing double-blind clinical trial. At the present time, they are available from the corresponding author on reasonable request. Once the study is unblinded, the datasets will be made publicly available.

## References

[1] Dettman SJ (2016). Long-term communication outcomes for children receiving cochlear implants younger than 12 months: A multicenter study. Otol. Neurotol. Off. Publ. Am. Otol. Soc. Am. Neurotol. Soc. Eur. Acad. Otol. Neurotol..

[2] Geers AE, Strube MJ, Tobey EA, Pisoni DB, Moog JS (2011). Epilogue: Factors contributing to long-term outcomes of cochlear implantation in early childhood. Ear Hear..

[3] Hayes H, Geers AE, Treiman R, Moog JS (2009). Receptive vocabulary development in deaf children with cochlear implants: Achievement in an intensive auditory-oral educational setting. Ear Hear..

[4] Holt RF, Beer J, Kronenberger WG, Pisoni DB, Lalonde K (2012). Contribution of family environment to pediatric cochlear implant users’ speech and language outcomes: Some preliminary findings. J. Speech Lang. Hear. Res..

[5] Leigh JR, Dettman SJ, Dowell RC (2016). Evidence-based guidelines for recommending cochlear implantation for young children: Audiological criteria and optimizing age at implantation. Int. J. Audiol..

[6] Niparko JK (2010). Spoken language development in children following cochlear implantation. JAMA.

[7] Bouton S, Serniclaes W, Bertoncini J, Colé P (2012). Perception of speech features by French-speaking children with cochlear implants. J. Speech Lang. Hear. Res..

[8] Nittrouer, S. & Caldwell-Tarr, A. Language and literacy skills in children with cochlear implants: Past and present findings. in *Pediatric Cochlear Implantation* 177–197 (2016).

[9] Tinnemore, A. R., Zion, D. J., Kulkarni, A. M., Chatterjee, M. Children’s recognition of emotional prosody in spectrally degraded speech is predicted by their age and cognitive status. *Ear Hear.* **2018 Jan 1**, (2018). 10.1097/AUD.0000000000000546 PMC6046271 29337761

[10] Gifford RH (2018). The relationship between spectral modulation detection and speech recognition: Adult versus pediatric cochlear implant recipients. Trends Hear..

[11] Jung KH (2012). Psychoacoustic performance and music and speech perception in prelingually deafened children with cochlear implants. Audiol. Neurotol..

[12] Lee KY, van Hasselt CA, Chiu SN, Cheung DM (2002). Cantonese tone perception ability of cochlear implant children in comparison with normal-hearing children. Int. J. Pediatr. Otorhinolaryngol..

[13] Olszewski C, Gfeller K, Froman R, Stordahl J, Tomblin B (2005). Familiar melody recognition by children and adults using cochlear implants and normal hearing children. Cochlear Implants Int..

[14] Peng SC, Tomblin JB, Cheung H, Lin YS, Wang LS (2004). Perception and production of mandarin tones in prelingually deaf children with cochlear implants. Ear Hear..

[15] Yeung HH, Werker JF (2009). Learning words’ sounds before learning how words sound: 9-Month-olds use distinct objects as cues to categorize speech information. Cognition.

[16] Olsho LW (1985). Infant auditory perception: Tonal masking. Infant Behav. Dev..

[17] Spetner NB, Olsho LW (1990). Auditory frequency resolution in human infancy. Child Dev..

[18] Hall JW, Grose JH (1991). Notched-noise measures of frequency selectivity in adults and children using fixed-masker-level and fixed-signal-level presentation. J. Speech Hear. Res..

[19] Allen P, Wightman F (1994). Psychometric functions for children’s detection of tones in noise. J. Speech Hear. Res..

[20] Buss E, Hall JW, Grose JH, Dev MB (1999). Development of adult-like performance in backward, simultaneous, and forward masking. J. Speech Lang. Hear. Res..

[21] Leibold LJ, Neff DL (2007). Effects of masker-spectral variability and masker fringes in children and adultsa). J. Acoust. Soc. Am..

[22] Leibold LJ, Werner LA (2006). Effect of masker-frequency variability on the detection performance of infants and adultsa). J. Acoust. Soc. Am..

[23] Nozza RJ, Wilson WR (1984). Masked and unmasked pure-tone thresholds of infants and adults: Development of auditory frequency selectivity and sensitivity. J. Speech Hear. Res..

[24] Oh EL, Wightman F, Lutfi RA (2001). Children’s detection of pure-tone signals with random multitone maskers. J. Acoust. Soc. Am..

[25] Werner LA, Bargones JY (1991). Sources of auditory masking in infants: Distraction effects. Percept. Psychophys..

[26] DiNino M, Arenberg JG (2018). Age-related performance on vowel identification and the spectral-temporally modulated ripple test in children with normal hearing and with cochlear implants. Trends Hear..

[27] Noble AR (2023). Spectrotemporal modulation discrimination in infants with normal hearing. Ear Hear..

[28] Buss E, Porter HL, Leibold LJ, Grose JH, Hall JW (2016). Effects of self-generated noise on estimates of detection threshold in quiet for school-age children and adults. Ear Hear..

[29] Allen P, Wightman F (1992). Spectral pattern discrimination by children. J. Speech Hear. Res..

[30] Jahn KN, Arenberg JG, Horn DL (2022). Spectral resolution development in children with normal hearing and with cochlear implants: A review of behavioral studies. J. Speech Lang. Hear. Res. JSLHR.

[31] Kirby BJ, Browning JM, Brennan MA, Spratford M, McCreery RW (2015). Spectro-temporal modulation detection in children. J. Acoust. Soc. Am..

[32] Peter V (2014). Assessing spectral and temporal processing in children and adults using temporal modulation transfer function (TMTF), Iterated Ripple Noise (IRN) perception, and spectral ripple discrimination (SRD). J. Am. Acad. Audiol..

[33] Rayes H, Sheft S, Shafiro V (2014). Discrimination of static and dynamic spectral patterns by children and young adults in relationship to speech perception in noise. Audiol. Res..

[34] Landsberger DM, Padilla M, Martinez AS, Eisenberg LS (2018). Spectral-temporal modulated ripple discrimination by children with cochlear implants. Ear Hear..

[35] Nittrouer S, Lowenstein JH, Sinex DG (2021). The contribution of spectral processing to the acquisition of phonological sensitivity by adolescent cochlear implant users and normal-hearing controls. J. Acoust. Soc. Am..

[36] Gifford RH, Hedley-Williams A, Spahr AJ (2014). Clinical assessment of spectral modulation detection for adult cochlear implant recipients: A non-language based measure of performance outcomes. Int. J. Audiol..

[37] Saoji AA, Litvak L, Spahr AJ, Eddins DA (2009). Spectral modulation detection and vowel and consonant identifications in cochlear implant listeners. J. Acoust. Soc. Am..

[38] Drennan WR, Anderson ES, Won JH, Rubinstein JT (2014). Validation of a clinical assessment of spectral-ripple resolution for cochlear implant users. Ear Hear..

[39] Henry BA, Turner CW (2003). The resolution of complex spectral patterns by cochlear implant and normal-hearing listeners. J. Acoust. Soc. Am..

[40] Won JH, Drennan WR, Rubinstein JT (2007). Spectral-ripple resolution correlates with speech reception in noise in cochlear implant users. J. Assoc. Res. Otolaryngol. JARO.

[41] Gifford RH, Noble JH, Camarata SM, Sunderhaus LW, Dwyer RT, Dawant BM, Dietrich M, Labadie RF (2018). The relationship between spectral modulation detection and speech recognition: Adult versus pediatric cochlear implant recipients. Trends Hear..

[42] Horn DL (2017). Effects of age and hearing mechanism on spectral resolution in normal hearing and cochlear-implanted listeners. J. Acoust. Soc. Am..

[43] Davidson LS, Geers AE, Uchanski RM (2021). Spectral modulation detection performance and speech perception in pediatric cochlear implant recipients. Am. J. Audiol..

[44] Nittrouer S, Lowenstein JH (2015). Weighting of acoustic cues to a manner distinction by children with and without hearing loss. J. Speech Lang. Hear. Res..

[45] Landsberger DM (2019). Temporal modulation detection in children and adults with cochlear implants: Initial results. Otol. Neurotol. Off. Publ. Am. Otol. Soc. Am. Neurotol. Soc. Eur. Acad. Otol. Neurotol..

[46] Tuz D, Aslan F, Böke B, Yücel E (2020). Assessment of temporal processing functions in early period cochlear implantation. Eur. Arch. Otorhinolaryngol..

[47] Blankenship C, Zhang F, Keith R (2016). Behavioral measures of temporal processing and speech perception in cochlear implant users. J. Am. Acad. Audiol..

[48] Blankenship CM, Meinzen-Derr J, Zhang F (2022). Within- and across-frequency temporal processing and speech perception in cochlear implant users. PloS One.

[49] De Ruiter AM, Debruyne JA, Chenault MN, Francart T, Brokx JPL (2015). Amplitude modulation detection and speech recognition in late-implanted prelingually and postlingually deafened cochlear implant users. Ear Hear..

[50] Fu Q-J (2002). Temporal processing and speech recognition in cochlear implant users. Neuroreport.

[51] Winn MB, Won JH, Moon IJ (2016). Assessment of spectral and temporal resolution in cochlear implant users using psychoacoustic discrimination and speech cue categorization. Ear Hear..

[52] Holder JT, Dwyer NC, Gifford RH (2020). Duration of processor use per day is significantly correlated with speech recognition abilities in adults with cochlear implants. Otol. Neurotol. Off. Publ. Am. Otol. Soc. Am. Neurotol. Soc. Eur. Acad. Otol. Neurotol..

[53] Cohen J (1988). Statistical Power Analysis for the Behavioral Sciences.

[54] Holder JT, Sheffield SW, Gifford RH (2016). Speech understanding in children with normal hearing: Sound field normative data for babyBio, BKB-SIN, and QuickSIN. Otol. Neurotol..

[55] Sheffield SW, Haynes DS, Wanna GB, Labadie RF, Gifford RH (2015). Availability of binaural cues for pediatric bilateral cochlear implant recipients. J. Am. Acad. Audiol..

[56] Gifford RH, Olund AP, DeJong M (2011). Improving speech perception in noise for children with cochlear implants. J. Am. Acad. Audiol..

[57] Teagle HFB, Park LR, Brown KD, Zdanski C, Pillsbury HC (2019). Pediatric cochlear implantation: A quarter century in review. Cochlear Implants Int..

[58] Viemeister NF (1979). Temporal modulation transfer functions based upon modulation thresholds. J. Acoust. Soc. Am..

[59] Walker BA, Gerhards CM, Werner LA, Horn DL (2019). Amplitude modulation detection and temporal modulation cutoff frequency in normal hearing infants. J. Acoust. Soc. Am..

[60] Cabrera L, Varnet L, Buss E, Rosen S, Lorenzi C (2019). Development of temporal auditory processing in childhood: Changes in efficiency rather than temporal-modulation selectivity. J. Acoust. Soc. Am..

[61] Nittrouer S, Lowenstein JH (2023). Asynchronies in auditory and language development obscure connections to phonological deficits in children. Am. J. Audiol..

[62] Jung KH (2012). Psychoacoustic performance and music and speech perception in prelingually deafened children with cochlear implants. Audiol. Neurootol..

[63] Roid GH, Miller LI, Pomplun M, Koch C (2013). Leiter-3: Leiter International Performance Scale.

[64] Levitt H (1971). Transformed up-down methods in psychoacoustics. J. Acoust. Soc. Am..

[65] Peterson GE, Lehiste I (1962). Revised CNC lists for auditory tests. J. Speech Hear. Disord..

[66] Uhler K, Warner-Czyz A, Gifford R (2017). Pediatric minimum speech test battery. J. Am. Acad. Audiol..

[67] Spahr AJ (2014). Development and validation of the pediatric AzBio sentence lists. Ear Hear..

[68] BKB-SIN Test. Etymotic Research Inc. (2005).

[69] Nilsson M, Soli SD, Sullivan JA (1994). Development of the Hearing in Noise Test for the measurement of speech reception thresholds in quiet and in noise. J. Acoust. Soc. Am..

[70] Compton-Conley CL, Neuman AC, Killion MC, Levitt H (2004). Performance of directional microphones for hearing aids: Real-world versus simulation. J. Am. Acad. Audiol..

[71] Gifford RH (2013). Cochlear implantation with hearing preservation yields significant benefit for speech recognition in complex listening environments. Ear Hear..

[72] Gifford RH, Dorman MF (2019). Bimodal Hearing or Bilateral Cochlear Implants?. Ask the Patient. Ear Hear..

[73] Studebaker GA (1985). A ‘rationalized’ arcsine transform. J. Speech Hear. Res..

[74] West, S. G., Finch, J. F. & Curran, P. J. Structural equation models with nonnormal variables: Problems and remedies. in *Structural Equation Modeling: Concepts, Issues, and Applications* 56–75 (Sage Publications, Inc, 1995).

