# Supplementary material for: The impact of spectral and temporal processing on speech recognition in children with cochlear implants
Source: Sci Rep. 2024 Jun 18;14:14094. doi: 10.1038/s41598-024-63932-w (PMC11189542; doi:10.1038/s41598-024-63932-w)
Supplement: Supplementary file 1 — Supplementary Information. [file 41598_2024_63932_MOESM1_ESM.docx]

**Supplementary Figure 1**

***Spectral and Temporal Resolution Thresholds by Age Group***


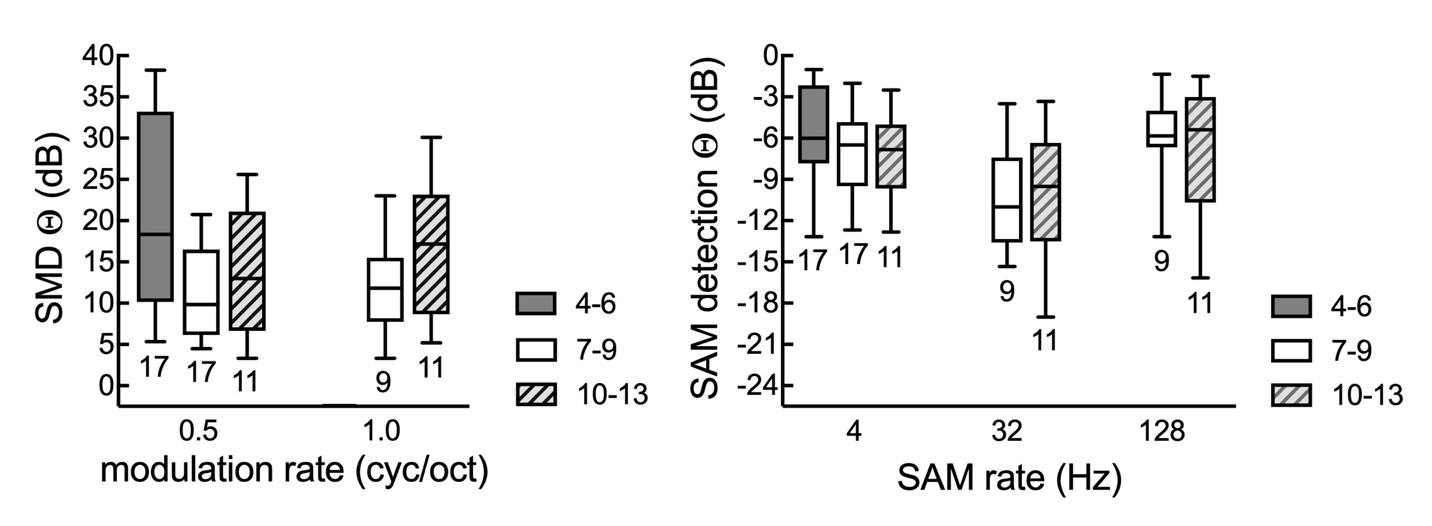


*Note***.** Spectral (SMD) and temporal (SAM) resolution thresholds (Θ) were subdivided for three age groups: 4-6, 7-9, and 10-13 year olds. Sample sizes for each group are listed below each box and whisker plot. The box represents interquartile range, the horizontal line represents median, and the whiskers extend to the 5^th^ and 95^th^ percentile.
